# Supplementary material for: Diversity Analysis and Function Prediction of Bacterial Communities in the Different Colored Pericarp of Citrus reticulata cv. ‘Shatangju’ Due to ‘Candidatus Liberibacter asiaticus’ Infection
Source: Int J Mol Sci. 2023 Jul 14;24(14):11472. doi: 10.3390/ijms241411472 (PMC10380603; doi:10.3390/ijms241411472)
Supplement: Supplementary file 1 [file ijms-24-11472-s001.zip › Supplementary Figure and Table S2.pdf]

Supplementary Table S2. Statistical analysis of microbial Alfa-diversity conducted by Simpson Index and Shannon index

Treatment	Comparison	*p*-value
Simpson Index		
WDYF	WGF	0.0005001***
WDYF	TYBGF	0.00005997***
WDYF	WLYF	0.00000006676***
WLYF	WGF	0.003796**
WLYF	TYBGF	0.4283
TYBGF	WGF	0.03757*
Shannon index		
WDYF	WGF	0.0002765***
WDYF	TYBGF	0.000007224***
WDYF	WLYF	0.00001467***
WLYF	WGF	0.008492**
WLYF	TYBGF	0.5369
TYBGF	WGF	0.009984**

Note: *** $p < 0.001$; ** $p < 0.01$; * $p < 0.05$

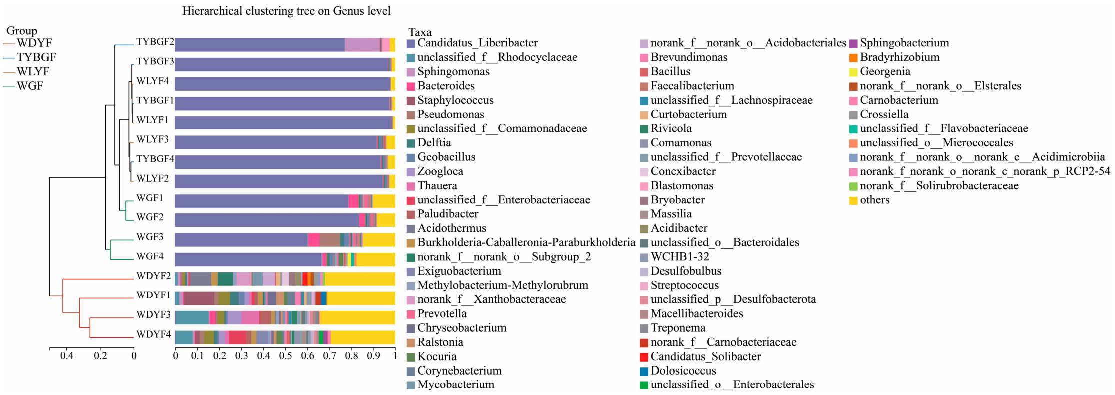

Supplementary Figure S1. Hierarchical clustering tree of bacterial in four types of pericarp samples

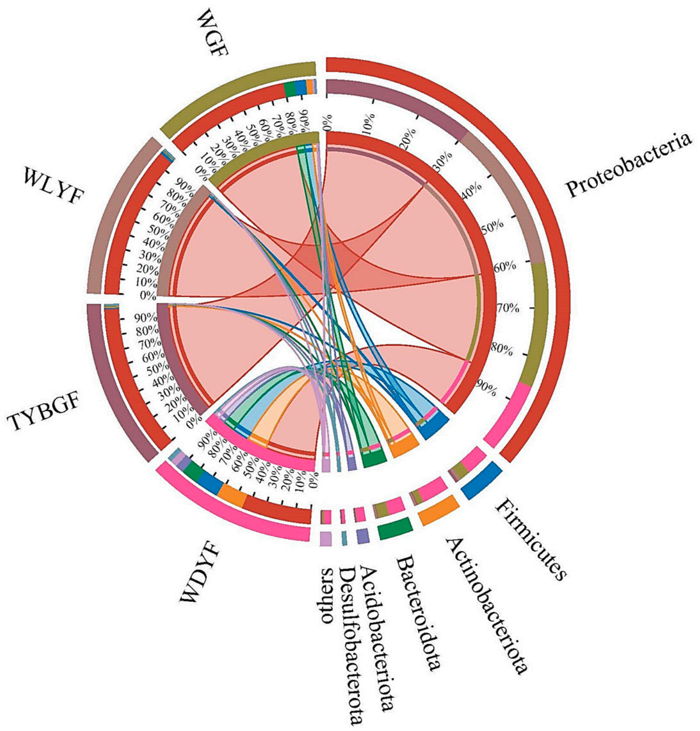

Supplementary Figure S2. Circos plot of bacterial composition in four types of pericarp samples

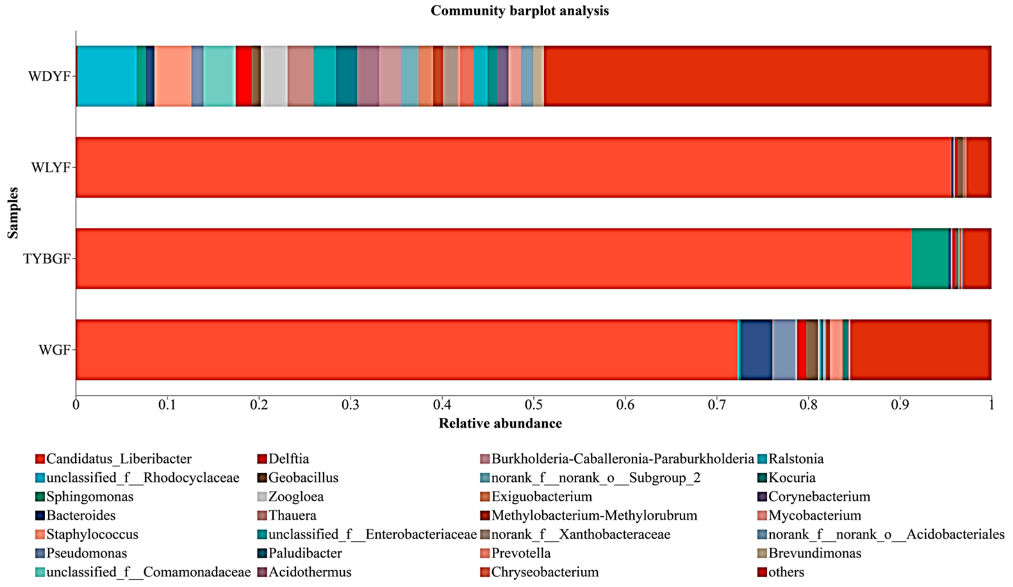

Supplementary Figure S3. The community bar plot analysis of bacterial composition in four types of pericarp samples

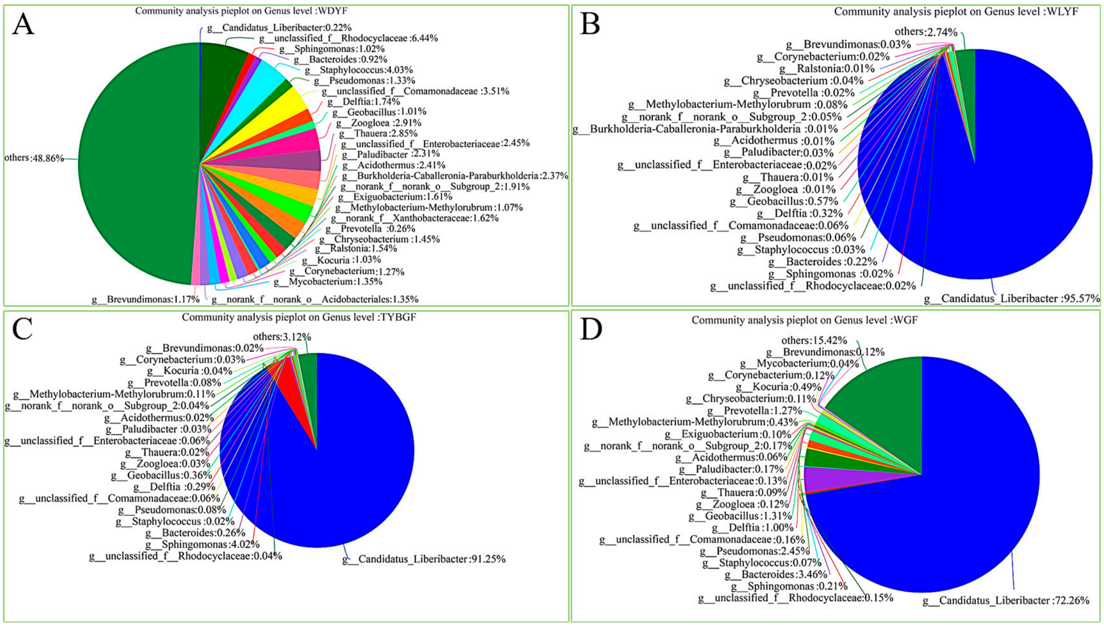

Supplementary Figure S4. Bacterial genera composition in the four types of pericarps. (A) Microbial community pie plot of bacteria in WDYF. (B) Microbial community pie plot of bacteria in WLYF. (C) Microbial community pie plot of bacteria in TYBGF. (D) Microbial community pie plot of bacteria in WGF.

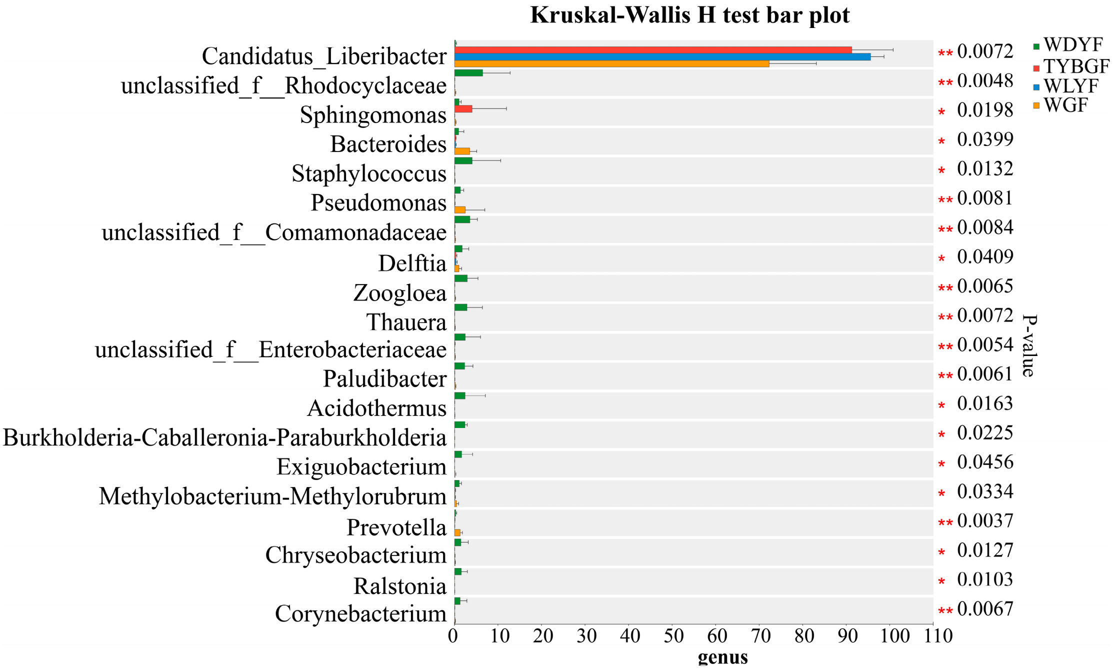

Supplementary Figure S5. Kruskal-Wallis H test bar plot analysis of the relative proportions of major genera. *p*-values were calculated using the Kruskal–Wallis H test. *** $p < 0.001$; ** $p < 0.01$; * $p < 0.05$

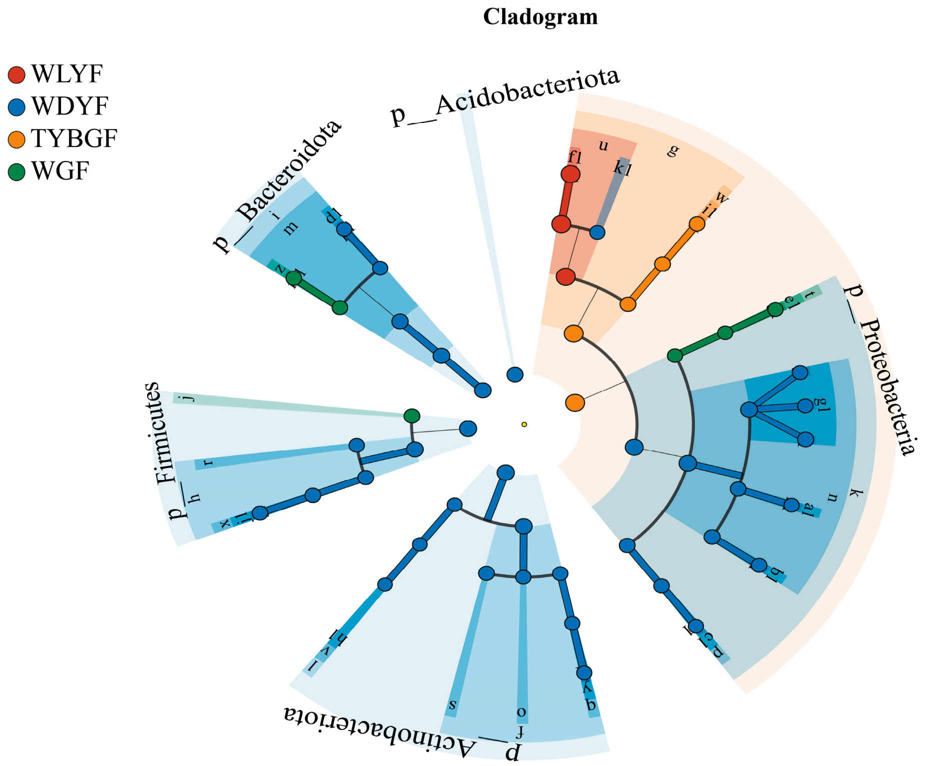

- a : p__Acidobacteriota
- b : p__Actinobacteriota
- c : p__Bacteroidota
- d : p__Firmicutes
- e : p__Proteobacteria
- f : c__Actinobacteria
- g : c__Alphaproteobacteria
- h : c__Bacilli
- i : c__Bacteroidia
- j : c__Clostridia
- k : c__Gammaproteobacteria
- l : c__Thermoleophilia
- m : o__Bacteroidales
- n : o__Burkholderiales
- o : o__Corynebacteriales
- p : o__Enterobacterales
- q : o__Frankiales
- r : o__Lactobacillales
- s : o__Micrococcales
- t : o__Pseudomonadales
- u : o__Rhizobiales
- v : o__Solirubrobacterales
- w : o__Sphingomonadales
- x : o__Staphylococcales
- y : f__Acidothermaceae
- z : f__Bacteroidaceae
- a1 : f__Burkholderiaceae
- b1 : f__Comamonadaceae
- c1 : f__Enterobacteriaceae
- d1 : f__Paludibacteraceae
- e1 : f__Pseudomonadaceae
- f1 : f__Rhizobiaceae
- g1 : f__Rhodocyclaceae
- h1 : f__Solirubrobacteraceae
- i1 : f__Sphingomonadaceae
- j1 : f__Staphylococcaceae
- k1 : f__Xanthobacteraceae
- l1 : g__Acidothermus
- m1 : g__Bacteroides
- n1 : g__Burkholderia-Caballeronia-Paraburkholderia
- o1 : g__Candidatus_Liberibacter
- p1 : g__Paludibacter
- q1 : g__Pseudomonas
- r1 : g__Sphingomonas
- s1 : g__Staphylococcus
- t1 : g__Thauera
- u1 : g__Zoogloea
- v1 : g__unclassified_f__Comamonadaceae
- w1 : g__unclassified_f__Enterobacteriaceae
- x1 : g__unclassified_f__Rhodocyclaceae

Supplementary Figure S6. Taxonomic cladogram obtained from linear discriminant analysis effect size analysis of 16S sequences in four types of pericarp samples. The circles from inner to outer represent the taxonomic levels of phylum, class, order, family, genus, and species.

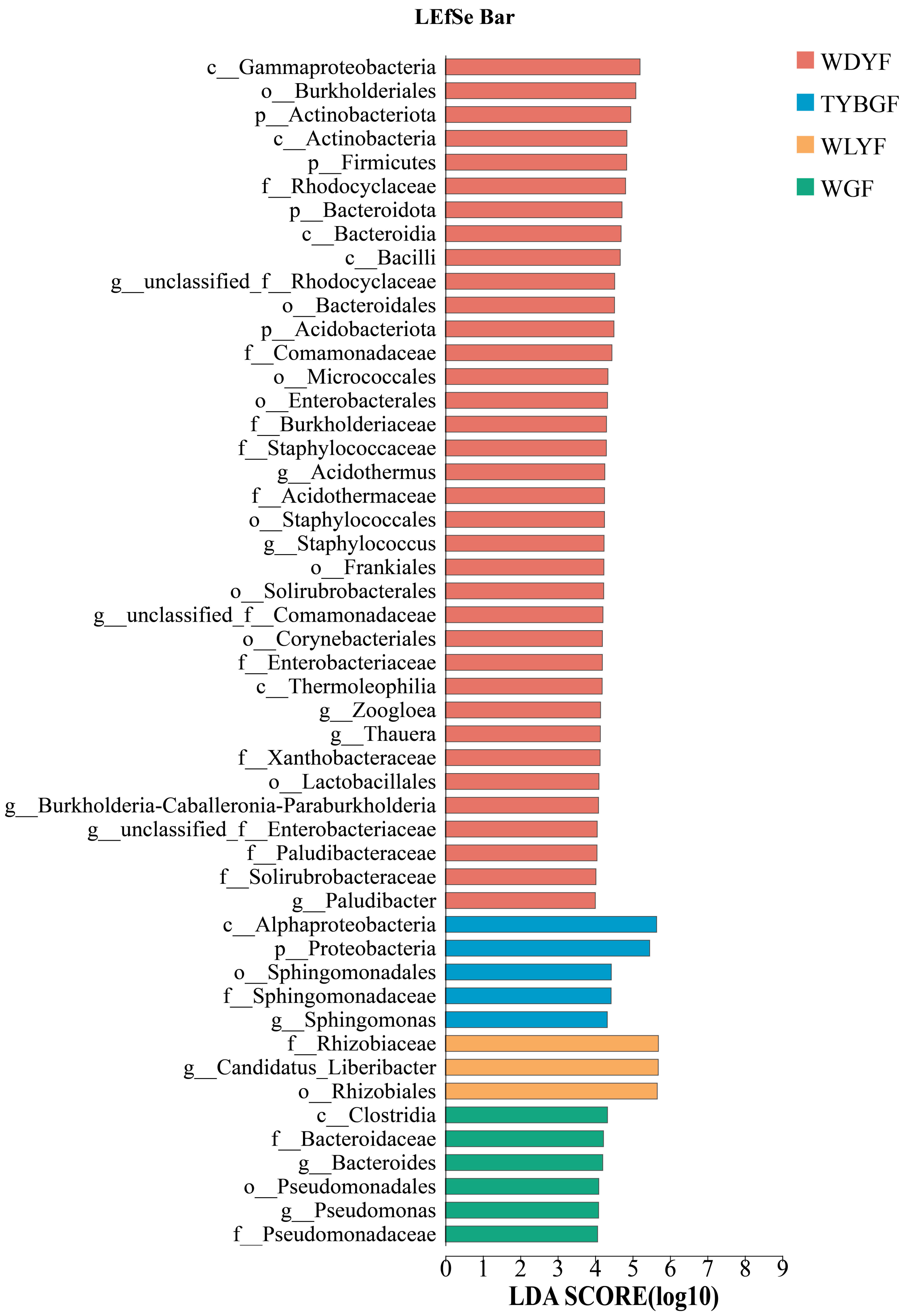

Supplementary Figure S7. Linear discriminative analysis (LDA) score of differentially enriched bacteria obtained from LEfSe analysis of microbiota from phylum to genus in four types of pericarp samples

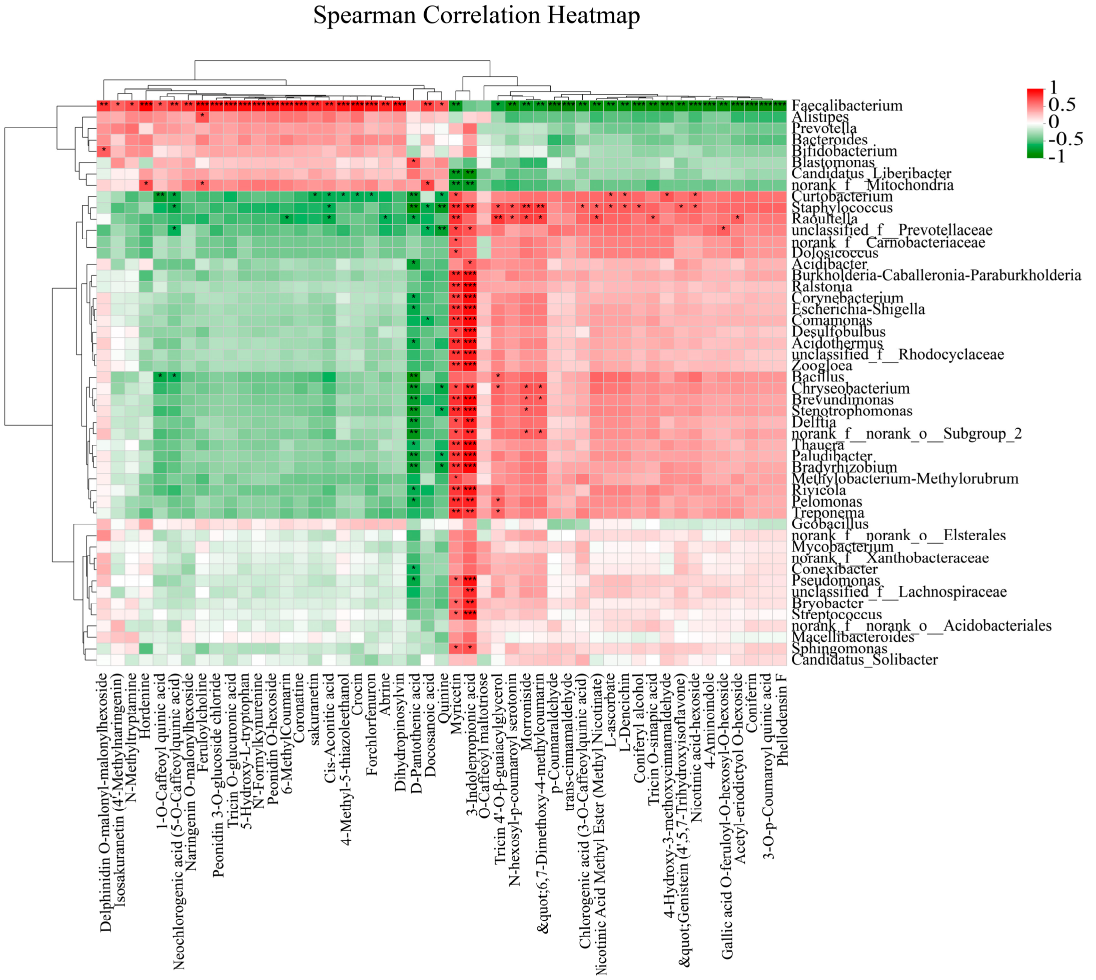

Supplementary Figure S8. Correlation analysis of metabolites and bacterial genus in four types of pericarp samples. *** $p < 0.001$; ** $p < 0.01$; * $p < 0.05$

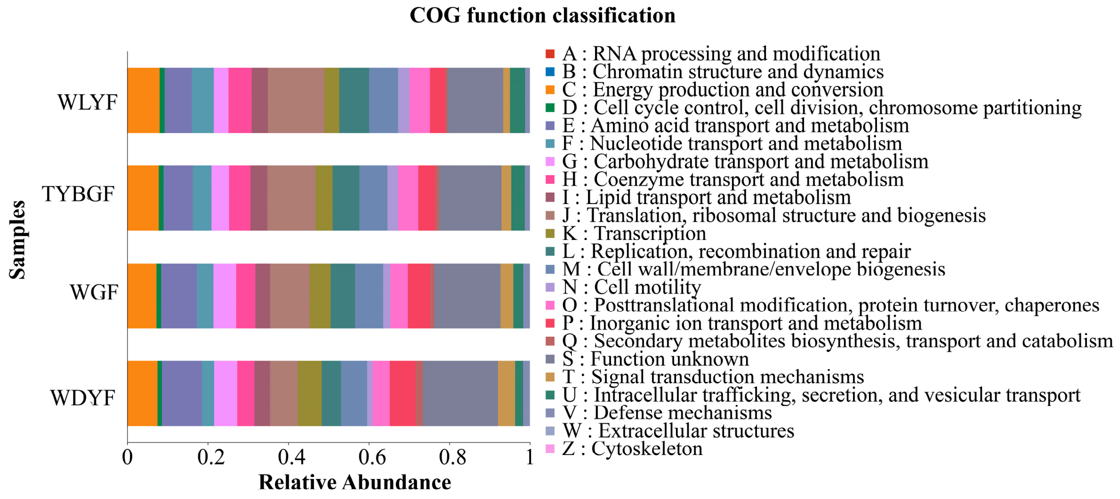

Supplementary Figure S9. The COGs function predicted for 16S rRNA of the citrus pericarp by PICRUST

Figure7. Significant difference in function predicted for KEGG pathway level_3, *, adjust *p*-value <0.05; **, adjust *p*-value <0.01; ***, adjust *p*-value <0.001. ko01100: Metabolic pathways; ko01110: Biosynthesis of secondary metabolites; ko01120: Microbial metabolism in diverse environments; ko01230: Biosynthesis of amino acids; ko02010: ABC transporters; ko03010: Ribosome.

Figure 8. Significant difference in function predicted for KO (A and B), and Enzyme (C and D). *, adjust *p*-value <0.05; **, adjust *p*-value <0.01; ***, adjust *p*-value <0.001. K03088: RNA polymerase sigma-70 factor, ECF subfamily; K01990: ABC-2 type transport system ATP-binding protein; K01992: ABC-2 type transport system permease protein; K06147: ATP-binding cassette, subfamily B, bacterial; K01952: phosphoribosylformylglycinamidine synthase; K00058: D-3-phosphoglycerate dehydrogenase/2-oxoglutarate reductase; K07391: magnesium chelatase family protein; K00526: ribonucleoside-diphosphate reductase beta chain; K16079: outer membrane immunogenic protein; K02651: pilus assembly protein Flp/PilA; 2.7.7.7: DNA-directed DNA polymerase; 3.6.4.12: DNA helicase; 2.7.13.3: Histidine kinase; 1.6.5.3: NADH:ubiquinone reductase (H(+)-translocating); 5.2.1.8: Peptidylprolyl isomerase; 2.7.7.6: DNA-directed RNA polymerase; 6.3.5.7: Glutaminyl-tRNA synthase (glutamine-hydrolyzing); 6.3.5.6: Asparaginyl-tRNA synthase (glutamine-hydrolyzing); 6.4.1.2: Acetyl-CoA carboxylase; 3.6.3.14: H(+)-transporting two-sector ATPase; 1.17.4.1: Ribonucleoside-diphosphate reductase; 6.3.5.3: Phosphoribosylformylglycinamidine synthase; 1.1.1.95: Phosphoglycerate dehydrogenase.
